# Genetic Contributions of Inflammation to Depression

**DOI:** 10.1038/npp.2016.169

**Published:** 2016-10-26

**Authors:** Jacob Barnes, Valeria Mondelli, Carmine M Pariante

**Affiliations:** 1 Department of Psychological Medicine, Institute of Psychiatry, Psychology and Neuroscience, King's College London, London, UK

## Abstract

This paper describes the effects of immune genes genetic variants and mRNA expression on depression's risk, severity, and response to antidepressant treatment, through a systematic review on all papers published between 2000 and 2016. Our results, based largely on case–control studies, suggest that common genetic variants and gene-expression pathways are involved in both immune activation and depression. The most replicated and relevant genetic variants include polymorphisms in the genes for interleukin (IL)-1β, IL-6, IL-10, monocyte chemoattractant protein-1, tumor necrosis factor-alpha, C-reactive protein, and phospholipase A2. Moreover, increased blood cytokines mRNA expression (especially of IL-1β) identifies patients that are less likely to respond to conventional antidepressants. However, even for the most replicated findings there are inconsistent results, not only between studies, but also between the immune effects of the genetic variants and the resulting effects on depression. We find evidence that these discrepant findings may be explained, at least in part, by the heterogeneity of the depression immunophenotype, by environmental influences and gene × environment interactions, and by the complex interfacing of genetic variants with gene expression. Indeed, some of the most robust findings have been obtained in patients developing depression in the context of treatment with interferon-alpha, a widely used model to mimic depression in the context of inflammation. Further ‘omics' approaches, through GWAS and transcriptomics, will finally shed light on the interaction between immune genes, their expression, and the influence of the environment, in the pathogenesis of depression.

## Introduction

Depression presents a clinical puzzle, affecting individuals in a multitude of differing ways (Pariante and Nemeroff, 2012). Antidepressants can treat depression, but with varying efficacy (Pigott *et al*, 2010). The condition is both hereditary and environmental (Sullivan *et al*, 2000). The biological mechanisms behind these discordant features remain elusive, frustrating our ability to address it. A solution may come from an interplay between the immune system and depression (Pollak and Yirmiya, 2002; Zunszain *et al*, 2011).

Some depressed patients express higher levels of cytokines, the messengers of the immune system, whereas the administration of cytokines themselves can cause depression. Many studies have reported increased levels of inflammatory cytokines and their receptors in the peripheral blood and cerebrospinal fluid of patients with major depression. In addition, patients with major depression have been found to exhibit elevations in peripheral blood concentrations of acute phase proteins, chemokines, adhesion molecules, and inflammatory mediators such as prostaglandins. These findings have been reviewed extensively before (Raison *et al*, 2006; Dantzer *et al*, 2008; Liu *et al*, 2012; Valkanova *et al*, 2013; Miller and Raison, 2015).

One hypothesis posits that this co-occurrence of depression and inflammation derives from the need for a rapid, preemptive inflammatory response following stress, which was life-saving during ancestral times when infection was often fatal (Miller and Raison, 2015). Inflammation may also have occurred when an individual felt threatened by another, because that physical attack may open up a route for infection. These ancestral mechanisms are visible in the fact that both depression and immune activation can result from social conflict, through activation of the inflammasome complex (Iwata *et al*, 2013; Maslanik *et al*, 2013). Indeed, the behavioral features of depression, like social withdrawal, anorexia, anhedonia, and hypervigilance, may themselves help the immune system to mount a response to infection, and cytokines induces the so-called ‘sickness behavior', a correlate of the inflammatory response (Peters, 2006). Taking this further, depression may be a chronic, over-extended version of sickness behavior. Indeed, in the developed world medical science has largely contained the infection challenge; yet this inbuilt defensive combination of depression and inflammation is still with us, like redundant heavy artillery left lying around from an earlier conflict—unwanted, perhaps dangerous, and hard to shift. One potential explanation for this continuing combination is that similar genes are relevant to both immune activation and depression.

Demonstrating a common genetic substrate for depression and immune activation is of substantial scientific and clinical interest, and could unveil the mechanisms by which the innate immune system and depression intertwine. Indeed, twin studies have shown a hereditable link between depression and inflammation (Su *et al*, 2009). In this review we look at the inflammatory genes that shape the nature of depression and, in particular, the polymorphisms that accentuate or attenuate this process. Although no genetic magic bullet has been found—no clearly responsible single-nucleotide polymorphism (SNP) such as in sickle cell disease—research suggests that individual polymorphisms have a role in the susceptibility to the degree of, and the recovery from, depression (Caspi *et al*, 2003; Manuck *et al*, 2004). Studies have also begun to unravel the plastic nature of inflammatory genes, particularly the influence of adverse childhood events on the immune system and later susceptibility to depression (Baumeister *et al*, 2015). More recently, genome-wide association studies (GWAS) have been employed to elicit candidate genes for depression. GWAS have had some spectacular results in other pathologies such as diabetes and rheumatoid arthritis (Hardy and Singleton, 2009), but have had limited success so far with depression. Finally, gene-expression studies and transcriptomics have begun to complement and supplant GWAS in whole-genome investigation. Gene-expression studies have begun to trace out networks of genes with links to inflammation involved in depression pathogenesis, and improving technology, such as RNA-seq, have allowed wider and more sensitive investigations than the previous gold-standard gene-expression microarrays (Hepgul *et al*, 2013). A new picture of networks and pathways connecting behavior and the immune system is being uncovered.

## Materials and methods

This review encompasses the literature published between 2000 and 2016. We limited our review to these years to best showcase current thinking about the genetic relationship between immune activation and depression, and to include studies employing the most up-to-date methods. We build on, and extend, our previous review on this topic (Bufalino *et al*, 2013). We focus on the relationship between genetic polymorphisms of immune genes and depression's risk, severity, and response to antidepressant treatment, including major depressive disorder (MDD), recurrent depression, dysthymia, childhood-onset major depression, and geriatric depression. In addition, we have included studies examining the prevalence of depression in subjects with medical illnesses, such as cardiac diseases, cancer, and those receiving cytokine therapy like interferon-alpha (IFN-*α*).

The literature reviewed was identified through the following sources: PubMed, The Cochrane Library, Scopus Embase, Ovid of Medline, PsycINFO, and ISI web of Knowledge. We considered case–control, prospective, twin/family-based association, and GWASs. In addition we included pharmacogenetic studies to better understand the mechanisms involved in the relationship between immune activation and depression in relation to antidepressant response, and gene-expression studies as both candidate genes and transcriptomics. Keywords included the following: ‘gene' or ‘genes' or ‘single nucleotide polymorphisms' or ‘SNPs' ‘depression' or ‘depressive disorder' and ‘inflammation' or ‘inflammatory cytokine' or ‘interleukin' or ‘interferon-a treatment' or ‘CRP'. Papers concerning animal models were excluded. The search was also limited to English-language studies. In total, we examined 113 studies and 4 meta-analyses. Numerous studies have tested more than one genetic variant, and are repeatedly cited.

## Results

We begin with pro- and anti-inflammatory cytokine genes, T-cell function genes, and C-reactive protein (*CRP*) gene. We then examine genetic variations in enzymes involved in immune activation and oxidative stress. Finally, we examine results from GWAS and gene-expression studies.

### Interleukin-1-beta

The interleukin-1-beta (*IL-1β*) gene is highly polymorphic, with the -511 C/T variation (rs16944) a particular target of study. The first study of the -511T polymorphism was conducted by Yu *et al* (2003a) who found that a subgroup of MDD patients homozygous for the ‘low-producing' 511C allele had higher depressive symptom severity and a less-favorable response to fluoxetine treatment when compared with 511T carriers. Tadic *et al* (2008) then demonstrated that the C/C genotype showed a slower and less-pronounced response to paroxetine than patients with the C/T or T/T genotype. No association was found between the 511T gene variant and mirtazapine treatment response (Tadic *et al*, 2008). In a Chinese population (Hwang *et al*, 2009), depressed patients carrying the C/C genotype showed a significantly earlier age of onset of depression compared with depressed subjects who were C/T heterozygous or homozygous for the T allele. Similar findings were also reported in dysthymic patients, who had a higher prevalence of the 511C allele compared with controls (Fertuzinhos *et al*, 2004).

In contrast, an association was found between the ‘high-producing' T variant and depressive symptoms in subjects with Alzheimer's disease (McCulley *et al*, 2004) and schizophrenia (Rosa *et al*, 2004). Kim *et al* (2013a) found the T/T form of IL-1β -511 to be associated with depression at baseline and 1-year follow-up in breast cancer patients. However, in a Taiwanese sample, this was not associated with MDD (Chi *et al*, 2011).

The variance of these polymorphic effects in the 511 SNP may depend on exposure to early life stress. A study of 1053 caucasian Hungarians volunteers (Kovács *et al*, 2016a) found that the presence of the A allele of rs16944 (equivalent to T 511), when paired with childhood maltreatment, leads to increased depressive and anxiety symptoms in adult life. However, the same SNP conferred a weak protective effect against depressive symptoms (but not anxiety) when the individual was exposed to life stressors as an adult (and had not experienced childhood maltreatment). A paper by Tartter *et al* (2015) examined the 511 variations in a study of 444 Australian youths. The C allele was associated with greater depressive symptoms following interpersonal stress. The same result was not found following exposure to other stressors, such as negative health and work events, and the depressive symptoms did not occur until after 20 years of age, leading the authors to theorize that the genetic effects of exposure to adverse childhood events only manifest in late adolescence.

Recently, in addition to 511(C/T), a polymorphism in the promoter region of the *IL-1β* gene at position 31 (T-31C substitution, located in a TATA-box motif of IL-1β) has been investigated in patients with major recurrent depression. Correspondence analysis revealed that a combination of genotype T/T for polymorphic site 31 and genotype C/C at position 511 was associated with depression, whereas controls presented a heterozygous combination at both polymorphic sites (Borkowska *et al*, 2011). Instead, no clear evidence has been found in childhood depression (Misener *et al*, 2009; 2008) or geriatric depression (Luciano *et al*, 2010). In a study of 599 Caucasian lung cancer patients, Reyes-Gibby *et al* (2013a) found an association between *IL-1β* T-31C and a symptom cluster of pain, depressed mood, and fatigue.

In a sample of 256 MDD patients, three SNPs located within regulatory regions of the *IL-1β* gene were investigated: 3953 C/T (rs1143634) in exon 5, rs1143643(A/G) in intron 6, and the aforementioned rs16944(A/G) in the promoter region. In addition, this study also evaluated whether any of these SNPs were associated with antidepressant treatment response and neural substrates of emotion processing. They found a significant association between the G/G genotype of rs16944 (same as CC 511) and G/G genotype of rs114643 with non-remission after 6 weeks of antidepressant treatment. Interestingly, functional magnetic resonance imaging showed that, in a subgroup of patients, the same genetic variants were associated with reduced responsiveness of the amygdala and the anterior cingulate cortex to emotional stimulation (Baune *et al*, 2010). The 3953 C/T (rs1143634) polymorphism was investigated also in subjects with post-stroke depression, but with negative findings (Kim *et al*, 2011). A paper on 773 elderly Koreans (Kang *et al*, 2014) also found a significant association between *IL-1β* 3953 C/T and depression, however, this association was lost once a Bonferroni correction was applied. In another study, the *IL-1β* SNP rs1143643 did not enhance depressive symptoms when combined with childhood trauma. However, the minor A allele did provide protection against depression due to trauma in adult life (Kovács *et al*, 2016a). This same allele was previously associated with a better response to antidepressant therapy (Baune *et al*, 2010).

Finally, Ridout *et al* (2014) examined 198 ethnically diverse American children ages 3–5 years exposed to maltreatment in the preceding 6 months. They found an association between rs1143633 AA genotype and greater symptoms of MDD when exposed to contextual stressors, such as loss, instability, or poverty. However, the study was limited by having only a small number of children homozygous for AA.

### IL-1 receptor antagonist

In two different studies, no significant associations were found between childhood depression (Misener *et al*, 2008), or antidepressant response to paroxetine and mirtazapine in MDD patients (Tadic *et al*, 2008), and IL-1 receptor antagonist (IL-1R*a*; 86 bp) polymorphism. Only one study has found that this polymorphism confers a susceptibility to develop dysthymia in Brazilian patients (Fertuzinhos *et al*, 2004).

### IL-1 receptor, type I

Doong *et al* (2014) found no association between *ILR1* and a symptom cluster of pain, sleep disturbance, fatigue, and depression.

### IL-1 receptor type 2

A haplotype (A1) consisting of three SNPs (rs4141134, rs11674595, and rs7570441) of IL-1 receptor type 2 (*IL-1R2*) was found to be associated with subsyndromal depression in a study of 167 oncology patients and caregivers in the US (Dunn *et al*, 2013). IL-1R2 is a decoy cytokine receptor that binds IL-1B. The study theorized that reduced expression of IL-1R2 leads to an excess of IL-1B, accounting for depressive symptomatology.

### IL-4

Kim *et al* (2011) have substantially investigated this anti-inflammatory IL. They found a significant association between post-stroke major depression and the ‘low-producing' C/C genotype of the +33T/C *IL-4* polymorphism (Kim *et al*, 2011), but no association among breast cancer patients and depression (Kim *et al*, 2013a). Moreover, they found that subjects with the +33C allele showed a relationship between increasing numbers of physical disorders and incident late-life depression (Kim *et al*, 2013b).

Illi *et al* (2012) found evidence for the involvement of the rs2443248 (T/G) SNP among a group of oncology patients, with the minor G allele associated with worse depression, fatigue, sleep, and pain.

### IL-6

Il-6 has been one of the most investigated ILs due to its high association with depression (Liu *et al*, 2012; Valkanova *et al*, 2013), though this association is not without dispute (Chocano-Bedoya *et al*, 2014). IL-6 is secreted largely by monocytes and macrophages peripherally, and by astrocytes and microglia in the CNS. In several different studies, no significant associations were found between MDD, childhood depression, or post-stroke depression, and either of the polymorphisms at position 634 or at position 174 of the *IL-6* gene (Clerici *et al*, 2009; Hong *et al*, 2005; Misener *et al*, 2008, 2009). However, in a prospective study in patients receiving pegylated IFN-*α* and ribavirin treatment for hepatitis C virus (HCV) infection, Bull *et al* (2009) found that the functional G-174C polymorphism (rs1800795) in the promoter region of the *IL-6* gene predicted depressive symptoms but not fatigue. Carrying two copies of the ‘high-IL-6' G allele polymorphism was associated with a marked increase in depressive symptoms compared with the ‘low-IL-6' C allele (Bull *et al*, 2009). This finding was later replicated by Udina *et al* (2013), who again found that carrying the CC genotype of rs1800795 is associated with less-severe IFN-*α*-induced depression.

On the contrary, Kovács *et al* (2016b) found that, in the presence of stressful life effects, the risk of depression was higher in those homozygous for the low-producing C allele (rs1800795). However, they caution this effect may be part of a wider haplotype, exerting its effects in a non-independent manner on gene expression. Tartter *et al* (2015) also found that CC carriers for IL-6 174 were more likely to experience depressive symptomatology following chronic interpersonal stress, but not other stressors. They also noted that 174G confers protection against inflammation in adolescence, but increases the risk for inflammation in adulthood, possibly indicating an age-dependent function. Roetker *et al* (2012) found that women homozygous (CC/GG) for rs1800795 had an increased risk of depression, but only in the presence of other risk genes.

In a study of 398 female breast cancer patients, Doong *et al* (2014) also reported an association between patients homozygous for the high-producing G allele of *IL-6* (rs2069845) and a symptom cluster of pain, fatigue, sleep disturbance, and depression, potentially occurring as an interaction between SNPs for *IL-13* and tumor necrosis factor-alpha (*TNF-α*). In another study of breast cancer patients (Saad *et al*, 2014), being homozygous for the G allele of rs2069840 was associated with subsyndromal depression.

### IL-8

IL-8 is involved in neutrophil recruitment. A polymorphism of the *IL-8* gene at position 251(T/A) was investigated in subjects with post-stroke depression, with negative findings (Kim *et al*, 2011). Kim *et al* (2013a) also investigated *IL-8*-251T/A in breast cancer patients and again found no association between either of the alleles and depression. The same research team later found a relationship between increasing numbers of physical disorders and incident late-life depression in those with the *IL-8* 251A variant in the aforementioned study (Kim *et al*, 2013b). Kang *et al* (2014), however, found no association between *IL-8* −251T/A and depression in a study of 732 elderly Koreans.

In an examination of a symptom cluster of pain, fatigue, and depression in lung cancer (Reyes-Gibby *et al*, 2013b), patients with the low-producing T/T polymorphism of *IL-8* 251 were more likely to experience severe depression, but less susceptible to pain or fatigue.

### IL-10

IL-10 is an anti-inflammatory cytokine. A study investigating a polymorphism at position 819 in *IL-10* found no evidence of an association with MDD (Jun *et al*, 2002). Similarly, no associations were found in another study looking at childhood depression and the 819 (C/T), 1082 (G/A), or 592 (C/A) polymorphisms (Misener *et al*, 2008); and no associations were found in patients with a single depressive episode, with or without stressful life events prior to onset (Haastrup *et al*, 2012).

On the other hand, in a study of patients affected by bipolar disorder (type I or II) or MDD, an association was found between the polymorphism at position 1082 (G/A) and MDD (Clerici *et al*, 2009). Specifically, the ‘low-IL-10 producing', A/A genotype, was significantly more frequent in MDD patients than in controls. Similar results were reported by Kim *et al* (2011) who found a significant association between post-stroke depression and the A allele-A/A genotype of the same polymorphism (Kim *et al*, 2011). A further study conducted in patients with end-stage renal disease also confirmed these findings (Holtzman *et al*, 2012).

No association was found between *IL-10* 1082 G/A alleles (rs1800896) and depression (Kang *et al*, 2014) in a study of 732 elderly Koreans. Similarly, 1082 G/A did not correlate with depression in breast cancer patients (Kim *et al*, 2013a) and did not affect the relationship between physical health and incident late-life depression (Kim *et al*, 2013b). Continuing the negative findings, 1082 G/A was not associated with late-onset depression in a study of elderly Brazilians (Torres *et al*, 2013). However, in a study of >900 elderly Americans (Rana *et al*, 2014), rs1800896 was found to be associated with optimism when present in a multi-gene locus with MAO-a and fibrinogen gamma chain SNPs.

Contradictory findings on the genetic association between depression and IL-10 have been found by analyzing SNPs of the genomic region of the *IL-10* gene cluster. Traks *et al* (2008) found that the frequency of the T–G–C haplotype, containing SNPs from *IL-20* and *IL-24* genes, is higher among patients with MDD compared with controls (Traks *et al*, 2008). However, Koido *et al* (2009) did not confirm these results in patients with a diagnosis of MDD and panic disorder. Instead, they found strong allelic and genotypic associations between both diagnoses and a polymorphism in the gene of an inhibitor of kappa light-polypeptide gene enhancer in B-cells kinase epsilon, with a stronger association with panic disorder (Koido *et al*, 2009).

*IL-10* rs1518111 was investigated in a study of 167 oncology patients and caregivers (Dunn *et al*, 2013) and the rare A/A configuration found to be associated with subsydromal depression. This non-coding SNP has an unknown function but has previously been linked with Behçet's Disease.

### IL-10 Receptor B

No association was found between the IL-10 Receptor B (*IL-10RB*) polymorphism rs2834167 and depression in lung cancer patients (Reyes-Gibby, 2013b), but Lys47Glu (rs2834167) was associated with a symptom cluster of pain, depressed mood, and fatigue when combined with other mutant alleles (Reyes-Gibby, 2013a).

### IL-11

An important genome-wide pharmacogenetic study, in a large sample of MDD patients treated with antidepressants, found that the major alleles of SNP rs1126757 in the gene encoding *IL-11* and of SNP rs7801617 in the *IL-6* gene (C and G, respectively) predicted a worse antidepressant response (Uher *et al*, 2010). Powell *et al* (2013a) found that carriers of the A allele of rs1126757 had greater reduction in IL-11 mRNA levels in response to escitalopram treatment, and that this reduction was associated with clinical response, thus indicating a potential mechanism by which genetic variants and gene expression interact in conferring a behavioral phenotype.

### IL-13

One study (Doong *et al*, 2014) among 398 breast cancer patients prior to surgery found that the A allele of *IL-13* (rs1295686) was associated with a symptom cluster of pain, fatigue, sleep disturbance, and depression. This anti-inflammatory cytokine has a well-established link with asthma.

### IL-17

In the same study as above (Doong *et al*, 2014) *IL-17* was not associated with the cluster.

### IL-18

Haastrup *et al* (2012) investigated polymorphisms of the *IL-18* gene in depressed patients with or without stressful life events prior to depression. The authors examined two promoter SNPs, at position 607 (G/T) and at position 137 (C/G). They found that the major alleles of both polymorphisms (G and C, respectively) increased the risk of depression in subjects with previous stressful life events, but not in those without. In addition, there was a tendency for higher plasma IL-18 levels among depressed patients who were G/G homozygous at position 607 and C/C at position 137.

### IL-28-beta

A polymorphism (rs1297860 C/T) in the IL-28-beta (*IL-28β*) gene, normally associated with viral response in HCV patients during IFN-*α* treatment, has been studied by Lotrich *et al* (2011) in relationship with psychiatric symptoms in patients taking IFN-*α* treatment. They investigated whether this polymorphism could be related to depression, fatigue, sleep problems, and/or changes in appetite. The C allele was associated with better viral clearance, loss of energy, worsened sleep, and a change in appetite, but not with depression (Lotrich *et al*, 2011).

### Colony-stimulating factor 2 receptor-beta

A recent study has tested the association between SNPs of the colony-stimulating factor 2 receptor-beta (*CSF2Rβ*) gene and three major mental disorders (bipolar affective disorder, schizophrenia, and MDD) in the Chinese Han population. Chen *et al* (2011) found that two haplotypes, composed of three SNPs (rs2284031, rs909486, and rs738149), were strongly associated with schizophrenia and MDD. The T–C–A haplotype represented a risk haplotype, whereas the C–T–G was a protective haplotype. In addition, the rs738149 SNP was significantly associated with MDD, and rs2284031 with both MDD and schizophrenia (Chen *et al*, 2011).

### IFN-gamma

Among many functions, IFN-γ activates indoleamine-2,3 dioxygenase (IDO), an enzyme that metabolizes the serotonin-precursor, tryptophan, and increases depressogenic tryptophan metabolites. The *IFN-γ* gene has a variable length CA repeat in its first intron. Myint *et al* (2013) found that individuals homozygous for the CA repeat allele 2 had higher levels of serum kynurenine at baseline, a by-product of tryptophan metabolism. Following treatment with a wide range of therapies (selective serotonin reuptake inhibitor antidepressant, psychological therapy, and electroconvulsive treatment) depressed individuals either homozygous or heterozygous for the same allele also had greater tryptophan breakdown and higher serum kynurenine.

Oxenkrug *et al* (2011) compared the *IFN-γ* +874 (T/A) polymorphisms in HCV patients with or without IFN-*α*-induced depression (rs2430561). The results demonstrated that the ‘high-producing' T allele increased the risk of IFN-*α*-induced depression (Oxenkrug *et al*, 2011). However, in another study, Clerici *et al* (2009) did not find any differences for genotype or allele distribution of +874 IFN-gamma in MDD patients (Clerici *et al*, 2009).

### IFN-gamma receptor 1

Saad *et al* (2014) reported an association between subsyndromal depression and the rare A allele of rs9376268 in breast cancer patients.

### IFN-*α* receptor

Examining the role of the IFN-*α* receptor (*IFNAR1*) gene in an American sample with HCV, one study found that the 5/5 or 5/14 genotype of a G/T repeat dinucleotide microsatellite polymorphism within the promoter region was associated with a larger increase in depressive scores during IFN-*α* treatment, and with a superior antiviral activity. Furthermore, only patients with the 5/14 genotype were found to show an association with increased somatic and neurovegetative symptoms (Yoshida *et al*, 2005).

### Monocyte chemoattractant protein-1

Monocyte chemoattractant protein-1 (MCP1) is known to attract peripheral monocytes to the brain, resulting in an inflammatory reaction; it is also known as chemokine (C-C motif) ligand 2 (CCL-2). Two case–control studies have investigated the role of the G-2518A polymorphism, and all have found that the A allele is associated with an increased risk of psychopathology. In a Korean population, subjects with the A allele were found to have an increased risk of developing both MDD and psychotic features compared with those with the G allele (Pae *et al*, 2004b). Moreover, in a clinical sample of 96 Italian outpatients with MDD and bipolar disorder, a higher frequency of the A/A genotype and of the A allele was observed in subjects affected by bipolar disorder; moreover, bipolar disorder subjects with the A/A genotype had a higher number of suicide attempts and more frequent psychotic symptoms (Altamura *et al*, 2010). Interestingly, the A allele is considered the ‘low-producing' (Rovin *et al*, 1999), and we have recently shown that low serum levels of MCP1 predict lack of response to antidepressants (Carvalho *et al*, 2013).

### Tumor necrosis factor-alpha

A Korean study demonstrated that the ‘high-producing' A allele and the A/A genotype of the G-308A *TNF-α* (rs1800629) polymorphism were significantly associated with an increased risk of MDD (Jun *et al*, 2003b). In another study, the *TNF-α* 308A allele was also found to be associated with post-stroke depression (Kim *et al*, 2011). However, opposite results were found by Clerici *et al* (2009) who observed a different allele distribution of G-308A *TNF-α* among a sample of 84 Italian outpatients affected by bipolar disorder or MDD. In particular, the percentage of A carrying subjects was lower in subjects with MDD (Clerici *et al*, 2009). Another study looking at individuals with late-life MDD found that subjects affected by MDD had a higher percentage of the G/G genotype than G/A genotype (Cerri *et al*, 2009). None of the patients were found to have A/A genotype. In a study of 167 oncology patients (Dunn *et al*, 2013) individuals with the rare A allele are less likely to suffer from clinically significant levels of depression. In yet another study, in patients with a single depressive episode with or without stressful life events prior to MDD, no involvement was found for the *TNF-α* SNPs 308 (G/A) and 238 (G/A) (Haastrup *et al*, 2012). This finding is in line with reports from Misener *et al* (2008) who found no significant association with childhood depression and these same *TNF-α* polymorphisms, or two others in the promoter region at positions 1031 (T/C) and 857 (C/T) (Misener *et al*, 2008). Among 444 Australian youth, *TNF-α* 308G/A was not found to moderate depressive symptoms following chronic interpersonal stress exposure (Tartter *et al*, 2015). Finally *TNF-α* 308G/A was not found to contribute to depression in breast cancer patients (Kim *et al*, 2013a). We identified one further study concerning the *TNF-α* A-308G polymorphism (rs1800629) in relation to IFN-*α*-induced depression: the authors found a significant association with labile anger and fatigue but not with depression (Lotrich *et al*, 2010).

A further polymorphism at position −850 (C/T) was investigated, in post-stroke depression with negative findings (Kim *et al*, 2011). The same team assessed 850 (C/T) in a later study (Kim *et al*, 2013a) and found an association between 850T and depression, though with deviation from the Hardy–Weinberg equilibrium. Kim *et al*, (2013b) also found, in the aforementioned study, that the relationship between increasing numbers of physical disorders and incident late-life depression is present in those with the 850T allele.

The less-well investigated *TNF-α* SNP rs1800610 was associated with a cluster of fatigue, sleep disturbance, and depression in breast cancer patients when carrying one or both of the rare T alleles (Doong *et al*, 2014). The SNP potentially worked in conjunction with IL-6 and IL-13 variations to manifest this symptom cluster.

Finally, a GWAS found support for the involvement of *TNF-α* rs769178 polymorphism. In a sample of 1738 MDD patients, 57 genes were identified and 92 SNPs mapped. The *TNF-α rs769178* was the only gene found to be related to depression, and it remained significant after correcting for multiple testing. However, the authors specified that given the large number of candidate SNPs and genes that were tested, even this significance may well be a false-positive (Bosker *et al*, 2010).

### Tumor necrosis factor-beta

Also known as lymphotoxin-alpha, one study investigated the role of the *TNF-β* gene polymorphism at position +252(G/A) in the first intron (chromosome 6) regarding susceptibility to MDD, but no associations were found (Jun *et al*, 2003a). However, Dunn *et al* (2013) found an association between the rare A allele of TNF-b rs2229094 and subsyndromal depression.

### TNF receptor 2

In another study of lung cancer patients (Reyes-Gibby *et al*, 2013a), TNFR2 Met196Arg (rs1061622) was associated with a symptom cluster of pain, depressed mood, and fatigue.

### T-cell function-related polymorphisms

The dysregulation of the ubiquitin–proteasome system, the main mechanism for protein catabolism, has been a recent and interesting development. A study by Wong *et al* (2008) found that two untranslated regions SNPs critical for antigen processing and T-cell differentiation in proteasome b4 subunit (PSMD4; rs2296840) and in T bet (TBX2; rs17244587), T and A, respectively, were significantly associated with MDD (Wong *et al*, 2008). Moreover, the same study described four further polymorphisms relevant to T-cell function that were associated with antidepressant response: rs2231449 in the CD3 antigen epsilon subunit (*CD3E*) gene, rs34095 in the protein kinase C substrate heavy chain (*PRKCSH*) gene, rs1043307 in the proteasome 26S non-ATPase subunit 9 (*PSMD9*) gene, and rs3809758 in the signal transducer and activator of transcription 3 (*STAT3*) gene, with the at risk allele being, respectively, A, T, G, and G. Wong *et al* (2008) also found that the A allele of *PSMD13* (rs3817629) was associated with response to fluoxetine in MDD. This SNP was further investigated by Minelli *et al* (2015) who found that each G allele increases the risk for treatment resistant depression. They did not find an association between the *PSMD9* SNP rs1043307 and treatment response, though they did note a small association between the A allele of rs1043307 and anxiety disorders in MDD patients.

### CRP-related polymorphisms

CRP meta-analysis have identified this protein's link with depression (Valkanova *et al*, 2013), though this remains a contentious result (Wium-Andersen *et al*, 2014).

Halder *et al* (2010) examined the effects of three common *CRP* polymorphisms, rs1417938(A/T), rs1800947(C/G), and rs1205(C/ T), on depressive symptomatology and circulating CRP levels in 868 healthy individuals. The authors generated three-locus haplotypes, and found the T–G–C haplotype to be associated with CRP levels and the A–G–T to show a marginal association. Neither single loci nor haplotypes were related to depressive symptoms. However, higher depression scores were positively associated with CRP levels among individuals with the A–G–T haplotype (Halder *et al*, 2010).

In a large sample of older men, a cross-sectional study by Almeida *et al* (2009) hypothesized that two further *CRP* polymorphisms, rs1130864(C/T) and rs1205(G/A), were associated with higher and lower plasma CRP levels, respectively, and may influence symptoms of depression. The results showed that 5% of participants had significant depressive symptoms, and these individuals indeed had higher serum concentrations of CRP. However, although the T allele of the rs1130864 SNP was associated with an increase in serum concentrations of CRP, it was not associated with an increase in the risk of depression. Instead, the rs1205(G/A) polymorphism, which was associated with lower concentrations of serum CRP, was associated with an increase in the risk of depression (Almeida *et al*, 2009). Of note, however, another study conducted in two elderly cohorts showed differing results. In one cohort assessed at age 70, the ‘high-producing' T allele of rs1130864 and the ‘low-producing' A allele of rs1205 were related with anxiety and neuroticism in women only; and in the second cohort, assessed at age of 87 years, the rs1800947 was found to be associated with depression (Luciano *et al*, 2010).

Two studies have shown that genetic variations in *CRP* are associated with depression in the context of the metabolic syndrome. In the study mentioned previously regarding the effects of variations in the *CRP* gene on the association between depression and circulating CRP, body mass index (BMI) was found to partially account for the moderating effects of the A–G–T haplotype on the association between depression and circulating CRP (Halder *et al*, 2010). This suggests that haplotypic variation in the CRP locus moderates an association of depressive symptoms with circulating CRP, and this is partially mediated by BMI. A longitudinal, population-based study demonstrated that adolescent emotional problems were strongly related to the metabolic syndrome among C/C homozygotes, but not among T allele carriers of the *CRP* rs1205 polymorphism (Gaysina *et al*, 2010).

Ancelin *et al* (2015) examined five variants of the *CRP* gene with gender-specific results. Women homozygous for the minor allele of rs1205 (TT) were more likely to suffer depression, but had lower circulating levels of CRP. The minor alleles of rs1130864 (TT) and rs1417938 (AA) were protective against depression in women, and were associated with lower levels of circulating CRP in men, but not women. In men, no SNP was associated with depression. The authors concluded that CRP is not a mediating factor between depression and inflammation, and cannot be used as a diagnostic biomarker for depression. They speculated that hormones may explain the difference in CRP levels between the sexes.

Rs1417938 was also investigated by Cicchetti *et al* (2015) who found higher levels of CRP among children who had recently experienced maltreatment and carried at least one of the minor A alleles. Finally, Michopoulos *et al* (2015) found that rs1130864 was associated with increased CRP serum levels in traumatized African-American individuals.

### Phospholipase A2

Two studies have investigated the potential role of the BanI polymorphism of the cytosolic phospholipase A2 (*cPLA2*) gene in conferring susceptibility to depressive disorder, although totaling three independent samples, and all found that the G variant increases the risk of psychopathology. In a Korean population, the G variant was associated with an increased risk of MDD (Pae *et al*, 2004a). More recently, Su *et al* (2010) have found that the same polymorphism influences the risk of developing IFN-*α*-induced depression in HCV patients. Specifically, the ‘at risk' G/G genotype was associated with higher severity of somatic symptoms of depression in patients with IFN-*α*-induced depression and in a replication sample of patients with MDD unrelated to cytokine treatment. Moreover, subjects with this genetic variant had lower eicosapentaenoic acid and docosahexaenoic acid (DHA) levels before and during IFN-*α* treatment (Su *et al*, 2010), indicating a possible mechanism by which genetic variants may increase the risk of depression by influencing circulating biomarkers.

### Cyclo-oxygenase 2

A polymorphism in the promoter region of the *COX-2*-encoding gene, characterized by a G-to-C transversion at position 765, has been correlated with recurrent depressive disorder, with the G/G homozygote and G allele increasing the risk of depression by 2.5-fold (Galecki *et al*, 2010a). This is in line with other findings of genetic variations of the rs4648308 polymorphism related with IFN-*α*-induced depression. Su *et al* (2014) found that the ‘at risk' A allele and the A/G genotype in the COX2 rs4648308 polymorphism significantly increased the risk of developing IFN-*α*-induced depression. In addition, the ‘at risk' A/G genotype was associated with lower DHA levels before and during IFN-*α* treatment (Su *et al*, 2010).

Mendlewicz *et al* (2012) found no association between COX-2 SNPs rs5275, rs20417, and resistance, response or remission to antidepressants.

### Myeloperoxidase (MPO)

To our knowledge, only one study has investigated the role of the Myeloperoxidase (*MPO*) gene (G-463A polymorphism) in the in susceptibility to recurrent depressive disorder. The findings showed that the presence of the 463G allele increased the risk of depression by 1.5-fold, whereas in those who are homozygous, the risk of depression development increases by 1.7-fold (Galecki *et al*, 2010b).

### Nitric oxide synthases

Genetic variants of both inducible nitric oxide synthase (*iNOS*) and neuronal *(n)NOS* genes have been related to an increased risk of developing depression. In one study, Galecki *et al* (2010c) found that the functional SNP 1026 (C/A), located in the promoter region of the human *NOS2A* gene, was significantly associated with depression risk in a Caucasian sample (Galecki *et al*, 2010c). Furthermore, the same research group found that the presence of the G/G homozygote of the *NOS2A* gene and of the T/T homozygote of nNOS increased the risk of depression (Galecki *et al*, 2011). However, in two previous studies, the functional polymorphism of the *nNOS* gene was not associated with MDD and antidepressant (fluoxetine/fluvoxamine) response in an Asian population (Okumura *et al*, 2010; Yu *et al*, 2003b). Gałecki *et al* (2012) investigated the *NOS2A* gene again and concluded that the variant is not functional, and was not related to expression, though there was an increased mRNA expression of iNOS associated with recurrent depressive disorder (see below).

Carriers of the minor NOS1 rs2682826 T allele had a higher probability of depression in a study of 763 southern Italians (Montesanto *et al*, 2013). Sarginson *et al* (2014) examined 1222 individuals undergoing financial stress and found 8 NOS1 SNPs to impact on levels of depression. The study also postulated a model that some risk alleles were protective under low-stress conditions but become risk factors when impacted by life-threatening events.

Kurrikoff *et al* (2012) found an association between the variants of the *NOS 1* gene first exon 1f variable number tandem repeat (long and short alleles) and depression, but effects varied according to environmental influence and gender. Cheah *et al* (2015) found an association between eight *NOS 1* adapter protein (*NOSAP1*) polymorphisms and depression in a study of 235 schizophrenics in Australia. This protein has previously been strongly associated with schizophrenia, due to downregulation of NO. Lawford *et al* (2013) also investigated 13 *NOS1AP* SNPs in 121 Vietnam male war veterans with a diagnosis of PTSD. They found that the GG genotype of rs386231 was associated with an increased severity of depression.

### Leukotriene A4 hydrolase

Genetic variants in the key enzyme involved in the leukotriene pathway, the leukotriene A4 hydrolase (*LTA4H*) gene, have been related to depression in subjects with coronary artery disease. Zhao *et al* (2009) found a significant protective effect of a novel haplotype in the *LTA4H* gene, named HapE, on coronary artery disease, and depression in women, but not men. HapE carriers tended to have a lower frequency of coronary artery disease and depression compared with HapE non-carriers. The authors indicate that about 7% of the association between depression and coronary artery disease severity was explained by HapE (Zhao *et al*, 2009).

### Adhesion molecule-related polymorphisms

Specific polymorphisms of genes related to endothelial dysfunction and platelet aggregation have been shown to influence depressive symptoms in cardiac patients. One intronic SNP marker, rs216873 within the vonWillebrand factor (*vWF*) gene, and markers within vascular cellular adhesion molecule 1, were found to be significantly associated with depressive symptoms, especially in women with coronary artery disease (McCaffery *et al*, 2009). This is the only study that we have identified which examines the role of adhesion molecule-related polymorphisms. A GWAS study (Kao *et al*, 2012) specifically looking at biological pathways found an association between the cell-adhesion pathway and MDD. Specifically, the top four significant pathways were long-term depression, calcium-signaling, arrhythmogenic right ventricular cardiomyopathy, and cell-adhesion molecules.

### Indoleamine-2,3-dioxygenase

We are including the *IDO* gene within the serotonin-related pathway because of the well-known link between immune-activation-induced IDO activation, reduction of tryptophan availability, and production of depressogenic tryptophan metabolites. IDO is activated by IFN-γ and TNF-*α*. We identified three studies concerning genetic variants of the *IDO* gene related to IFN-α-induced depression. One found that a polymorphism (rs9657182) in the promoter region of the indoleamine-2,3-dioxygenase 1 (*IDO1*) gene predicts the development of moderate or severe depressive symptoms in Caucasian but not in African-American subjects undergoing IFN-α therapy for HCV infection. Patients who carried the C/C genotype were more likely to exhibit moderate or severe depression at week 12 of IFN-α treatment compared with those with either the C/T or T/T genotypes (Smith *et al*, 2011). However, a second cross-sectional study, conducted in a Brazilian population, found no associations (Galvao-de Almeida *et al*, 2011). Utilizing the STAR*D cohort, Cutler *et al* (2012) discovered two *IDO* SNPs (rs2929115 and rs2929116) that were associated with response to treatment with citalopram.

### Genome-Wide Association Studies

Despite great success within other pathologies, GWAS analyses of MDD have struggled to produce results at the SNP level. This may be due to various factors, including heterogeneity of the condition (eg, nine accessory symptoms in DSM-5 covering various phenotypes), the need for very large sample sizes (10 000 or more cases and controls), influence of the environment on causality, and that phenotypes may be the result of the interaction of many risk SNPs, genes, or pathways, rather than individual polymorphisms. Nevertheless, GWAS studies have confirmed some evidence of involvement of immune genes in depression.

As mentioned above, Bosker *et al* (2010) conducted a GWAS study that found support for the involvement of *TNF-α* rs769178 polymorphism. In a sample of 1738 MDD patients, 57 genes were identified and 92 SNPs mapped; the *TNF-α rs769178* was the only gene found to be related to depression and that remained significant after correcting for multiple testing. Another GWAS (Bosker *et al*, 2011) identified *TNF-α*, dendritic nuclear protein-1 (*DCNP-1*) and neuropeptide Y (*NPY*) as candidate genes. However, a later GWAS (Ripke *et al*, 2012) reported no significant associations with common polymorphisms. Recently, Song *et al* (2013) identified five immune-associated candidate genes that may be associated with MDD: *ANPEP* (degradation of neurotransmitters and IL-8 regulation), *ENPEP* (regulation of growth and differentiation of early B-lineage cells), *PRDM1* (a protein that represses inteferon-β gene expression), *ZBTB32* (may regulate the differentiation and activation of helper T-cells), and *MMP8* (matrix metalloproteinase-collagen degradation). In one of the largest GWAS studies to date (Okbay *et al*, 2016) subjective well-being was associated with rs3756290, an SNP coding for interferon regulatory factor 1.

More recently attention has turned to pathway analysis within GWAS as a means to elucidate the causal mechanisms behind MDD. As mentioned above, Kao *et al* (2012) found that the cell-adhesion molecule pathway was one of the four most associated with MDD. They also found that both the *TNF* gene and the *IL-1β* gene were within three different significantly enriched pathways. More interestingly, the Cross-Disorder Group of the Psychiatric Genomics Consortium (2013) identified calcium channel signaling pathways involved in a wide psychiatric phenotype, covering autism spectrum disorder, attention-deficit hyperactivity disorder, bipolar disorder, MDD, and schizophrenia. Altered calcium signaling has been associated with a downregulation of TNF-*α* and inducible NOS (Raison and Miller, 2012).

### Gene-expression (mRNA) studies/transcriptomics

As discussed before (Hepgul *et al*, 2013), peripheral blood mRNA analyses are now a consolidated approach for biomarker discovery in mental health, also considering that about 80% of genes are co-expressed, and similarly modulated at mRNA levels, in peripheral blood cells and in brain tissues. A number of studies have used hypothesis-driven approach, with candidate immune genes measured in relationship with depression and antidepressant response, although most recent work has focussed on transcriptomics analyses, where immune-related pathways have figured prominently among the biological systems consistently identified.

#### Candidate gene approach

Gałecki *et al* (2012) examined the expression of four genes (*PTGS2*, *MPO*, *NOS2A*, *PLA2GA*) coding for COX-2, MPO, iNOS, and secretory phospholipase A2 type IIA, and found them all to be increased in patients with recurrent depressive disorder, underlining the role of oxidative and nitrosative stress in this condition.

Cattaneo *et al* (2013) examined 9 inflammatory genes for response to antidepressant treatment (escitalopram *vs* nortryptline) in the GENDEP randomized-controlled trial. Depressed patients overall had higher levels of IL-1β, IL-6, macrophage-inhibiting factor (MIF), and TNF-*α*, and lower levels of the anti-inflammatory cytokine, IL-4, compared with controls. Patients who were less responsive to antidepressants also have the highest levels of IL-1β, MIF, and TNF-*α*, and successful antidepressant response was associated with normalization of IL-6 levels. The ability of blood IL-1β and MIF mRNA levels to predict antidepressant response has been recently replicated in a second, independent sample, where gene expression was measured using ‘absolute' mRNA values, a reliable quantitation of number of mRNA molecules (Cattaneo *et al*, 2016).

Utilizing the GENDEP trial cohort, 86 genes in the inflammatory cytokine pathway were investigated by Powell *et al* (2013b). This showed no significant reductions in transcription of IL-1β, IL-6, TNF or MIF, or any other of the 86 genes investigated in the cytokine pathway, following escitalopram treatment for 8 weeks. They did find significant increase in transcription of the cell-transporter protein ABCF1 following treatment; this protein has been associated with regulation of inflammatory cytokines. Increase in ABCF1 transcription was also associated with treatment response. SSRI treatment response was also investigated by Mamdani *et al* (2011) who found that response to treatment with citalopram was correlated with an increase in interferon regulatory factor 7 (IRF7) expression, a transcription factor that regulates IFN-*α*. The authors also examined prefrontal cortex tissue from *post mortems* of men who had died during a depressive episode, and found decreased expression of IRF7.

Guilloux *et al* (2014) had two major findings: that genes and transcription factors for immune and inflammation functions were upregulated in MDD patients, and that a 13-gene model predicted non-remission following antidepressant treatment. This model was successfully applied in both their own and a validation cohort, and genes in this group included interferon-induced transmembrane protein 3 (IFITM3) and the T-cell surface glycoprotein CD3D. Another study found increased baseline TNF expression in MDD and BPD patients *vs* healthy controls; pathway analysis revealed a network centered around TNF expression. (Savitz *et al*, 2013).

Using both GWAS and a candidate gene approach, Hori *et al* (2016) found 317 upregulated genes in MDD patients that were enriched for a synaptic transmission pathway and a protein–protein interaction network. This pathway echoes some of the same calcium-signaling genes (*CACNA1B*, *CACNA1E*) found by the PGC study (*CACNA1C* and *CACNB2*) (Ripke *et al*, 2012). In candidate analyses, most important genes of Hori *et al* were *VAMP2* (vesicle-associated membrane protein 2), *CSGALNACT1* (chondroitin sulfate *N*-acetylgalactosaminyltransferase 1, transcript variant 2), and *CRHR2* (the corticotropin-releasing hormone receptor), which showed the largest difference between patients and control.

An opposing result was found by Belzeaux *et al* (2012) in a small study (16 patients, 13 controls) in which IL-1β and TNF transcription upregulation were linked to increased treatment response, and the authors suggested could be used as predictors of treatment response. Belzeaux *et al* (2014) went on to produce an intriguing case report of expression measurement, noting an interesting pattern of expression of TNF-*α* while monitoring a single episode of MDE from presentation to treatment and finally resolution, with TNF expression reduced during the episode but returning to baseline following treatment. They also found that this TNF-*α* expression is linked to a protein (S100A10) regulating cerebral serotonin-signaling. Mehta *et al* (2013) also found that increased TNF mRNA at baseline was associated with treatment response, but in this case it was response to the anti-inflammatory, infliximab. Important negatives were also found by Spijker *et al* (2010) in which LPS-stimulated blood gene expression was used to examine the difference between MDD patients and controls: both groups showed high expression of *TNF*, *IL-1*, *IL-6,* and *IL-10* genes in response to the insult, but with no significant difference between patients and controls.

Measuring monocyte-only expression, Carvalho *et al* (2014) found many inflammatory genes that were upregulated in MDD patients compared with controls, and that expression of MCP1 (CCL-2) and IL-1β were (negatively) correlated with serum levels. The study also reports that elevated IL-8 serum levels were correlated with reduced expression of the glucocorticoid receptor alpha mRNA, supporting the hypothesis of an interaction between the hypothalamus-pituitary-adrenal axis and immune system. They also found that serum IL-6, IL-8, and MCP1 levels were significantly increased in MDD patients compared with controls.

Some studies have again used hepatitis C patients following interferon-/ribavirin-associated depression as a model of inflammation-related depression. Pawlowski *et al* (2014) found that increased expression of several cytokines, including IL-8, IL-10, IL-12, TNF-alpha, and IFN-beta, were associated with depression.

#### Transcriptomics studies

Two genome-wide transcription studies (Morag *et al*, 2011; Oved *et al*, 2012) initially found the neural cell-adhesion molecule L-1-like protein (CHL-1) to be a potential biomarker for depression. In an analysis of 463 patients and 459 controls, Mostafavi *et al* (2013) detected a significant association between MDD and the IFN-*α*/-*β* signaling pathway, via the upregulation of IFN-stimulated gene factor 3 (ISGF3)-induced genes. These studies were followed by the largest gene-expression study to date (Jansen *et al*, 2015). They identified 13 clusters associated with MDD. The most statistically significant cluster involved genes enriched for the ‘IL-12-mediated signaling events' and the ‘natural killer cell-mediated cytotoxicity' pathways. Another significant cluster was enriched with the ‘signaling by interleukins' and ‘IL-6-mediated signaling events' pathways. Interestingly, they did not find a difference of expression between controls and cases for IL-6, CRP or TNF-*α* serum levels, but did find that one of the receptors of TNF-*α* (TNFRSF10C), MAPK14, the IL-6 receptor and STAT3, were all upregulated, confirming an enrichment with genes in the IL-6 signaling pathway. For the authors, the combination of upregulated inflammatory cytokine genes and downregulated NK cell transcription is a confirmation of the immune suppression/hyper-activation theory of MDD. In the same study, one gene was found to be statistically significant at the transcriptional level: DVL3, whose participation in the evolutionary ancient Wingless-related integration site may be related to hippocampal neurogenesis. Similar results were also found in the Young Finns study (Elovainio *et al*, 2015), which used gene-set enrichment analysis to examine immune/ inflammatory pathways associated with depression. The IL-1 pathway was the set most associated with depression, followed by the toll-like pathway, NEF protein pathway, the nuclear factor kB pathway, the kinase AKT pathway and the mature B-cell antigen receptor pathway. Finally, Hepgul *et al* (2016) found that plasma cytokines were not predictive of the development of depression, but mRNA gene expression was: in pathway analysis, the study found that oxidative stress, IL-1, IL-6 and IL-8 pathways are associated with the development of depression.

## Concluding Remarks

This review synthesizes the current literature on the association between genetic variants involved in immune activation and depression's risk, severity, and response to antidepressants. Understanding how genetic variants influence the immune system's contribution to the development of depression is important for the identification of vulnerable individuals, for establishing clinical biomarkers and for the development of new pharmaceutical treatments. Our results suggest that common genetic variants and gene-expression pathways are involved in both immune activation and depression, and we have highlighted some consistent findings across the literature. Of note, we have focussed our review on genes relevant to depression, but findings relevant to bipolar disorder, schizophrenia, and autism, when overlapping with depression, have also been reported. It is likely that immune gene variants may be similarly, or even more relevant, for these disorders, that have stronger genetic and neurodevelopmental influences compared with depression.

### Can we identify a consistent pattern of findings?

The most replicated and relevant genetic variants, together with their putative molecular mechanisms, are presented in Table 1, and include polymorphisms in the genes for IL-1β, IL-6, IL-10, MCP1, TNF-α, CRP, and PLA2. However, even for the most replicated findings there are inconsistent results, both between studies and between the behavioral changes and the putative immune mechanisms. For example, within the IL-1β polymorphism in the promoter region at position 511, the T/T genotype is considered to be associated with increased secretion of IL-1β compared with the C/C genotype (Pociot *et al*, 1992). However, only two studies found evidence for an association of the 511T allele with depressive symptoms (McCulley *et al*, 2004; Rosa *et al*, 2004), whereas three other studies found that the ‘low-IL-1β' C allele is associated with higher depressive symptoms severity or with earlier age of onset (Fertuzinhos *et al*, 2004; Hwang *et al*, 2009; Yu *et al*, 2003a). Similarly, the ‘high-TNF-α' 308A allele was reported as increased in Korean subjects with major depression or with post-stroke depression (Jun *et al*, 2003b; Kim *et al*, 2011), but in the Caucasian population is the ‘low-TNF-α' G allele that is associated with major depression in the elderly (Cerri *et al*, 2009). Similarly, the CRP rs1205 (G/A) polymorphism, and the A allele of the *MCP1* gene, are both the ‘low-producing' alleles, and are both associated with an increase in the risk of depression (Almeida *et al*, 2009; Pae *et al*, 2004b). The potential molecular and biological correlates of these discrepancies have been discussed extensively before (Bufalino *et al*, 2013), and it is possible that ‘*in vitro*' studies describing the function of a specific polymorphism do not always map onto real biological effects. Indeed, these same genetic variants associated with depression also increase the risk of inflammation-related metabolic disorders (see below). However, here we want to highlight three areas of recent research that may help explaining these discrepant findings.

First, these genetic mechanisms may be relevant only to a subtype of ‘inflammation-related' major depression, and thus are diluted when analyzed in large pool of depressed patients unselected for a specific immunophenotype. The recent study by Raison *et al* (2013) on the efficacy of infliximab on treatment resistant depression finds that only ~30–40% of depressed patients (based on the criteria) present with raised inflammatory markers (CRP) at baseline, and it is likely that the distribution of high-inflammation genetic variants will be different in these patients compared with those with no peripheral inflammation. Indeed, some of the most robust findings have been obtained in patients developing depression in the context of treatment with IFN-*α*, a widely used model to mimic depression in the context of inflammation (see Box 1).

Second, environmental and gene × environment interactions are likely to have a larger role in inflammation-related depression than purely genetic mechanisms. For example, previous studies and recent meta-analytical evidence show that a history of childhood trauma is associated with inflammation in adulthood even in the absence of depression (Baumeister *et al*, 2015). Moreover, a number of studies mentioned above have shown that the effects of some SNPs may only become evident in the presence of life stressors. For example, Kovács *et al* (2016a) found that the ‘high-IL-1β' T allele of rs16944 leads to increased depressive symptoms in adult life but only in individuals exposed to childhood trauma, whereas conferring a weak protective effect against depressive symptoms when the individual is exposed to adult life stressors. A paper by Tartter *et al* (2015) found instead that the ‘low-IL-1β' C allele is associated with greater depressive symptoms in adulthood following interpersonal stress in childhood. Similar gene × environment interactions have been described for the *TNF-α* and the *IL-18* genes.

Finally, mRNA gene-expression studies have shown more reliable associations with outcomes then studies of genetic variants, and indeed have been able to explain the effects of genetic variants. For example, Powell *et al* (2013a) found that carriers of the A allele of rs1126757 had greater reduction in IL-11 levels in response to escitalopram treatment, and that this reduction was associated with clinical response. Moreover, the association between increased IL-1β and MIF mRNA and lack of antidepressant response have been replicated in two independent samples (Cattaneo *et al*, 2013, 2016). This might be due to the fact that gene-expression measurements represent the status of a large number of neurobiological systems involved in depression. For example, IL-1β increases IDO activity and reduces neurogenesis in experimental models (Zunszain *et al*, 2012), is responsive to a range of antidepressant mechanisms, including nutritional interventions (Horowitz *et al*, 2014), and contributes to the activation of the inflammasome complex; instead, MIF promotes neuroplasticity and neuroprotective processes under physiological conditions, but it can also increase the production of pro-inflammatory cytokines under conditions of stress, and is modulated by glucocorticoids (Cattaneo *et al*, 2016). Therefore, just measuring two genes can give enough information to take the pulse of a host of neurobiological systems. Furthermore, -omics approaches through GWAS and transcriptomics may finally shed light on the interaction between immune genes, immune genes expression and stressful environment in the pathogenesis of depression.

### Are these genetic variants relevant for mental and physical health?

Immune genetic variants that increase the risk of depression are also likely to increase the risk of obesity, diabetes, and the metabolic syndrome, and perhaps are contributing to the overlaps between depression and these other medical conditions. For example, TNF-α has an important role in insulin governance, and excess secretion can result in insulin resistance; and the A allele of the aforementioned *TNF-α* 308 G/A polymorphism has also been associated with type 2 diabetes mellitus, particularly in Asian subjects (Zhao *et al*, 2013). The -174 G allele of the *IL-6* gene has been associated with the development of the metabolic syndrome and diabetes (Huth *et al*, 2006) as well as atherosclerosis (Chumaeva *et al*, 2014) in the context of chronic stress. The A allele of the -1082 polymorphism of the *IL-10* gene has been associated with insulin resistance and obesity (Tarabay *et al*, 2016). MCP1 is considered to be a major attractant for macrophages in obesity, and the A allele of the G-2518 polymorphism is also associated with increased risk of diabetes and its complications (Zhang *et al*, 2011). Therefore, immune genes variants that increase the risk of depression also have widespread effects on behavior and biological activity, beyond the simple defense against infection, and these effects may be physiologically positive or negative, depending on the input of environmental factors and the needs of the body. For example, short-term insulin resistance can be an aid to fighting infection, and the development of atherosclerosis may be the result of a metabolic system that above all else mitigates against hypoglycemia and its immediate life-threatening consequences, rather than hyperglycemia, whose deleterious effects are only seen after many years (Kitano *et al*, 2004).

**Figure 1:**
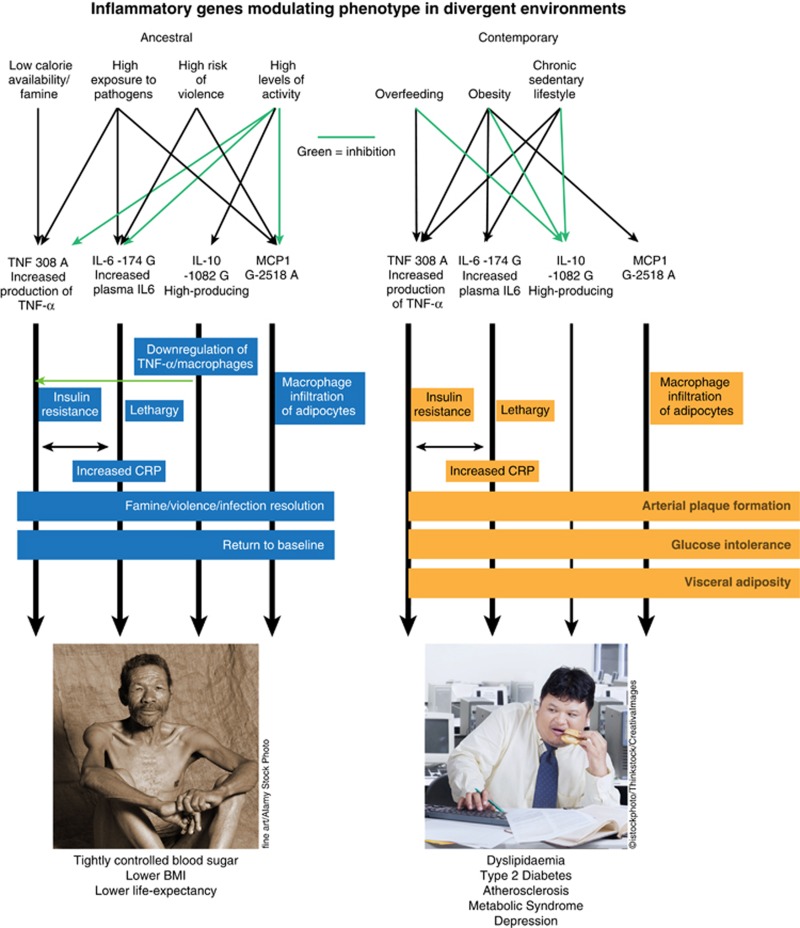
The conflicting needs of an individual according to the environment could explain why some immune genetic variants are still present in the human pool even if they confer risk of both depression and metabolic abnormalities. In a theoretical ancestral setting, ‘at risk' polymorphisms, such as the *TNF-α* 308A allele, may maintain plasma glucose in the harshest of conditions, allowing cerebral energy load to be maintained in the face of famine, acute stress (such as attack or flight), and infection. This polymorphism may also interact with the mutually reinforcing high-producing *IL-6* 174 G allele to mount a strong immune response to pathogens, and conserve energy via reduced physical activity during acute infection. Similarly, the *MCP1* 2518A allele reduces skeletal muscle uptake of glucose, making more energy available to the immune system for infection combat. At the same time, consistently high levels of activity employed in foraging and hunting help to keep the excesses of these polymorphisms in check, aided by the anti-inflammatory actions of the *IL-10* 1082 ‘protective' G polymorphism. However, in a modern setting, the sedentary lifestyle, the atmosphere of continuous low-level stress, and the tendency toward excess calorie intake, turn these genetic advantages into systemic millstones, creating a downward self-reinforcing, obesogenic, diabetic, pro-atherosclerotic system, whose ultimate result is the metabolic syndrome and cardiac disease.

These conflicting needs of an individual according to the environment could explain why some immune genetic variants are still present in the human pool even if they confer risk of both depression and metabolic abnormalities (see Figure 1). In a theoretical ancestral setting, ‘at risk' alleles, such as the *TNF-α* 308A allele, may maintain plasma glucose in the harshest of conditions, allowing cerebral energy load to be maintained in the face of famine, acute stress (such as attack or flight), and infection. This polymorphism may also interact with the mutually reinforcing high-producing *IL-6* 174 G allele to mount a strong immune response to pathogens, and conserve energy via reduced physical activity during acute infection. Similarly, the *MCP1* 2518A allele reduces skeletal muscle uptake of glucose, making more energy available to the immune system for infection combat. At the same time, consistently high levels of activity employed in foraging and hunting help to keep the excesses of these polymorphisms in check, aided by the anti-inflammatory actions of the protective *IL-10* 1082 G polymorphism.

However, in a modern setting, the sedentary lifestyle, the atmosphere of continuous low-level stress, and the tendency toward excess calorie intake, turn these genetic advantages into systemic millstones. Depression and stress activate the immune system, engaging these polymorphisms tendencies to raise blood sugars. Deprived of the anti-inflammatory effects of exercise, the *IL-10* 1082 G polymorphism loses its protective influence (and the A allele increases the risk of depression). Excess calorie intake creates more adipocytes, and the increased *TNF-α* production (via the 308A allele) creates insulin resistance, that in the short-term protects adipocytes from having to absorb excessive, toxic levels of glucose, but in the long-term leads to chronic elevated blood sugar and its diabetic consequences. Excess adipocytes also means excess MCP1, which recruit further macrophages to adipose tissue, which in turn secrete further TNF-α, creating a downward self-reinforcing, obesogenic, diabetic, pro-atherosclerotic system whose ultimate result is the metabolic syndrome and cardiac disease.

## Conclusions

Although it is difficult (and perhaps unwise) to wish for a return to an ancestral setting, nevertheless the powerful ability of the environment in shaping the effects of genetic variants is an important reminder that the trajectory of our mental and physical health is not deterministically defined. We believe that a framework linking stress, immune system and health, not only generates meaningful theoretical models, but also can be the focus of environmental interventions that bring together nutritional and psychosocial approaches with novel pharmaceutical tools.

## Funding and disclosure

This work was supported by the grants ‘Immunopsychiatry: a consortium to test the opportunity for immunotherapeutics in psychiatry' (MR/L014815/1) and ‘Persistent Fatigue Induced by Interferon-alpha: A New Immunological Model for Chronic Fatigue Syndrome' (MR/J002739/1), from the Medical Research Council (UK). Additional support has been offered by the National Institute for Health Research Mental Health Biomedical Research Centre at South London and Maudsley NHS Foundation Trust and King's College London. CMP and VM have received research funding from Johnson & Johnson as part of a program of research on depression and inflammation. In addition, CMP and VM have received research funding from the Medical Research Council (UK) and the Wellcome Trust for research on depression and inflammation as part of two large consortia that also include Johnson & Johnson, GSK, and Lundbeck. CMP has also received consultation fees from Eleusis Benefit Corporation. JB declares no conflict of interest.

## Figures and Tables

**Table 1:** Most Investigated Polymorphisms and Their Findings

Gene	Polymorphism	Potential mechanism	Findings	Sample size	Authors
IL-1β	rs16944	The C allele is the ‘low-producer' this may be mediated by strong linkage disequilibrium with a TATA-box polymorphism at position −31 from the the IL-1 gene which influences DNA/protein interactions.	The C allele has been associated with an earlier age of onset of depression, higher depression severity, greater depression when exposed to stress, dysthymia, and a lower response to SSRI treatment.	Six papers totaling 1733 patients and controls	Hwang *et al*, 2009; Yu *et al*, 2003a; Tadic *et al*, 2008; Fertuzinhos *et al*, 2004; Tartter *et al*, 2015; Baune *et al*, 2010
IL-6	rs1800795	This SNP is in the promoter region of the IL-6 gene. The G allele is associated with higher plasma concentration of IL-6 than the C allele. The G allele has been extensively linked with diabetes and heart disease.	Homozygosity for GG has been associated with greater depressive symptoms during interferon-alpha treatment. In breast cancer patients, it is linked with subsyndromal depression and a cluster of fatigue, pain, sleep disturbance, and depression. It has also been found to act differently according to age, mitigating inflammation in adolescence but increasing the chances of depression in adult life.	Four papers totaling 5738 patients	Bull *et al* 2009; Udina *et al* 2013;Roetker *et al* 2012; Tartter *et al* 2015
IL-10	-1082A	IL-10 is an anti-inflammatory cytokine, and inhibits the production of pro-inflammatory TH1 cytokines, such as IL-6 and IFN-γ. The A allele is considered to be ‘low-producing'.	The low-producing AA genotype is associated with major depressive disorder, any type of depression following-stroke, and depressive symptoms in patients with end-stage renal disease.	Three papers totaling 1015 patients and controls	Clerici *et al*, 2009; Kim *et al*, 2011;Holtzman *et al*, 2012
MCP1	G-2518A	Also known as chemokine (C-C motif) ligand 2 (CCL-2), MCP1 attracts peripheral monocytes to the brain, resulting in an inflammatory reaction. The A allele is considered the ‘low-producing'.	The A allele is associated with an increased risk of developing both MDD and bipolar depression, and with an increased risk of psychotic features and of suicide attempts.	Two papers totaling 461 patients and controls	Pae *et al*, 2004b; Altamura *et al*, 2010
TNF-α	rs1800629	The common G allele is a ‘low-producer' compared with the rarer AA genotype.	The GG genotype was associated with depression, major depression in the elderly, and depression within the context of bipolar disorder.	Three papers totaling 749 patients, caregivers, and controls	Clerici *et al*, 2009; Cerri *et al*, 2009; Dunn *et al*, 2013
CRP	rs1205	CRP is a marker of inflammation and its synthesis is driven by IL-6. The A allele has been correlated with lower circulating CRP levels.	The A allele has been associated with more severe depression, anxiety, and adolescent emotional difficulties.	Four papers totaling 7428 individuals	Halder *et al*, 2010; Almeida *et al*, 2009; Luciano *et al*, 2010; Ancelin *et al*, 2015
Phospholipase A2	BanI	PLA2 is a family of enzymes that liberate free fatty acids from phospholipids, which are in turn transferred to the arachidonic acid pathway and result in the production of pro- and anti-inflammatory eicosanoids. The GG genotype has been associated with higher PLA2 enzyme activity in platelets, and lower levels of circulating PUFAs.	Carriers of the G allele of the BanI polymorphism were more likely to suffer from depression (especially somatic symptoms) and experience depression during treatment with IFN-α.	Two papers comprising 3 distinct samples and totaling 361 Asian patients and controls	Pae *et al*, 2004a; Su *et al*, 2010

## Box

IFN-α-induced depression has been one of the most useful models with which to examine the effects of the inflammatory process on mood disorder, with one in four patients experiencing depression during treatment. IFN-α stimulates IFN-γ, which in turn induces IDO, an enzyme that converts tryptophan into the kynurenine pathway (leading to neurotoxic and depressogenic metabolites) rather than toward the production of serotonin. Interestingly, a number of immune genes have been described as associated with the risk of IFN-α-induced depression. Using this model, there is clear and consistent evidence that genetic variants associated with increased immune activation are also associated with increased risk of depression. For example, in the first such study, Yoshida *et al* (2005) find that a polymorphism (5/14 genotype) in the IFNAR1 interferon receptor that confers higher immunologic activity is associated with more depressive symptoms. Interestingly, the authors also find that this gene increases viral response to the IFN-α therapy, thus describing a genetic association between depression and increased immune clearance. Indeed, Lotrich *et al* (2011) investigated a polymorphism (rs1297860 C/T) in the *IL-28b* gene, normally associated with viral response, and found that the C allele is associated with better viral clearance and with worse somatic symptoms of depression. Oxenkrug *et al* (2011) found that carriers of a high-producing polymorphism of IFN-gamma (rs2430561) are more likely to suffer depression during IFN-*α* treatment. Smith *et al* (2011) found a polymorphism of the *IDO 1* gene (rs9657182) that increases the enzymatic activity and is also associated with depression. Su *et al* (2010) found that polymorphisms in the *COX2* (rs4648308) and *PLA2* (BanI) genes are associated with reduced levels of polyunsaturated fatty acids (PUFAs) during IFN-*α* treatment. PUFAs have been associated with protection against cytokine-induced depression, and therefore this study identifies a putative molecular mechanism through which genetic variants may indirectly increase inflammation-induced depression through a reduction in the endogenous anti-inflammatory PUFAs levels. Indeed, the same authors followed-up their research with a clinical trial where they administered PUFAs as oral supplement prophylactically before starting IFN-α, and were able to drastically delay the onset and reduce the overall rates of depression (Su *et al*, 2014). IFN-α also stimulates IL-6 production, and two independent studies found that a polymorphism (rs1800795) conferring increased IL-6 activity in response to an immune challenge is associated with more severe depressive symptoms (Bull *et al*, 2009; Udina *et al*, 2013).
